# Female endurance athletes show a reduced plasma cell‐free DNA response to all‐out and 3‐h cycling compared to male counterparts

**DOI:** 10.14814/phy2.70999

**Published:** 2026-07-03

**Authors:** Kira Enders, Moritz Dollsack, Daniela Schranner, Stephanie Bremer, Perikles Simon, Henning Wackerhage, Elmo W. I. Neuberger, Martin Schönfelder

**Affiliations:** ^1^ Department of Sports Medicine, Disease Prevention and Rehabilitation Johannes Gutenberg University Mainz Mainz Germany; ^2^ Department of Health and Sport Sciences Technical University of Munich Munich Germany; ^3^ Institute of Computational Biology Helmholtz Zentrum München Neuherberg Germany

**Keywords:** biomarker, circulating DNA, cycling, endurance exercise, exercise physiology, sex differences

## Abstract

This study investigated cell‐free DNA (cfDNA) kinetics during an all‐out and 3‐h cycling protocol, focusing on sex differences. Eighteen participants (9 women, 9 men) performed a VO_2max_ test on a cycling ergometer. One to four weeks thereafter, 17 participants completed 3‐h cycling at an intensity 5% below their first ventilatory threshold. Venous blood was drawn before and immediately after the VO_2max_ test, as well as before, during (every 30 min), and up to 24 h after the 3‐h cycling. cfDNA increased 4.7‐fold after the VO_2max_ test. After the 3‐h cycling, cfDNA increased 4.9‐fold and decreased slowly, reaching baseline after 24 h. Men showed higher cfDNA responses to exercise than women, which persisted after adjustment for energy expenditure relative to body mass. Non‐linear generalized additive models revealed significant sex differences in the relationship between cfDNA and energy expenditure per kg body mass (*β* = 0.61 ± 0.28, *t* = 2.22, *p* = 0.029) or lean body mass (*β* = 0.65 ± 0.27, *t* = 2.40, *p* = 0.019). These findings suggest sex‐specific factors, possibly including neutrophil activity and hormonal influences, contribute to cfDNA release during exercise and warrant further investigation into the underlying mechanisms.

## 1 INTRODUCTION

Circulating cell‐free DNA (cfDNA) is a potential marker to monitor inflammation, the severity of injury, and cellular stress in athletes (Huminska‐Lisowska et al., 2021), as well as exercise load (Atamaniuk et al., 2008; Breitbach et al., 2012). While cfDNA is already well established as a biomarker for several pathological conditions (Lo et al., 2021; Pessoa et al., 2020; Rahat et al., 2020), cfDNA may be a useful biomarker for training load or exercise‐induced microinjuries for several reasons. CfDNA responds within minutes after the onset of exercise (Tug, Mehdorn, et al., 2017) and peaks earlier compared to typical markers, like creatine kinase or uric acid (Breitbach et al., 2012). Furthermore, it has a longer biological half‐life, and additionally, already low‐level activity increases cfDNA concentration in the circulation (Haller et al., 2017). Previous research has indicated that increases in cfDNA are associated with neutrophil activation (Fatouros et al., 2006; Fridlich et al., 2023), thereby reflecting the inflammation and immune system activation triggered by exercise.

Various studies have already shown that cfDNA levels increase after acute exercise, depending on the modality (Neuberger, Hillen, et al., 2021), duration (Haller et al., 2017), and intensity (Beiter et al., 2011; Haller et al., 2017; Tug, Tross, et al., 2017) of exercise. In response to acute exercise, cfDNA levels increase up to 20‐fold (Atamaniuk et al., 2008), with running causing larger increases than cycling (Neuberger, Hillen, et al., 2021) or weightlifting (Atamaniuk et al., 2010; Fatouros et al., 2006; Tug, Tross, et al., 2017). After exercise, most studies reported a half‐life of the decline of cfDNA of about 15–25 min (Breitbach et al., 2012; Yamamoto et al., 2025) or a return to baseline within 2 h after the end of exercise (Atamaniuk et al., 2010; Sugasawa et al., 2021). However, Atamaniuk and colleagues observed 3.4‐fold elevated cfDNA concentrations 2 h after completing a six‐hour ultra‐marathon race (Atamaniuk et al., 2008).

To date, most studies on cfDNA and exercise have focused on male athletes only or have predominantly included men. Therefore, potential differences in the exercise‐induced changes of cfDNA concentrations between women and men remain underexplored. Recently, studies have started to address sex‐based differences in cfDNA in response to exercise (Blumkaitis et al., 2024; Nogiec et al., 2024) and examine the impact of the menstrual cycle on exercise‐induced cfDNA increases (Sawai et al., 2024). Blumkaitis et al. (2024) detected higher cfDNA concentrations in men after an all‐out running test. Nevertheless, according to differences in duration, and peak power output the observed differences cannot be conclusively attributed to sex. Additionally, Nogiec et al. (2024) measured significantly higher cfDNA concentrations in male compared to female participants in response to endurance exercise. However, this effect was no longer observed when normalizing for expended calories. Nogiec and colleagues argue that this is related to the small sample size of 6 men and 6 women. Thus, there is a lack of research into sex differences in the cfDNA response to exercise.

Therefore, the present study aimed to systematically investigate cfDNA kinetics in two distinct exercise protocols and to assess potential sex differences. The primary outcomes were (i) response of cfDNA concentrations to a graded cycle ergometer test to exhaustion when compared to a prolonged constant load ergometry test over 3 h in healthy endurance trained athletes, and (ii) sex differences in these responses. We hypothesized that both exercise protocols would induce significant increases in cfDNA. But we do not know whether there is a leveling off after prolonged endurance exercise. Furthermore, we expected distinct cfDNA kinetics between the graded exercise test to exhaustion and the 3‐h constant load cycling, reflecting their differing physiological demands. In addition, we tested the hypothesis that cfDNA responses differ between women and men, reflected by a significant time × sex interaction, given the emerging evidence for sex‐specific differences in cfDNA responses to exercise (Blumkaitis et al., 2024; Nogiec et al., 2024).

The secondary (exploratory) outcomes addressed the relationship between cfDNA responses and energy expenditure during the 3‐h constant load test, as well as potential sex‐specific differences in these associations. This was motivated by previous findings showing that exercise‐induced cfDNA changes are associated with exercise load and energy expenditure (Enders et al., 2025; Nogiec et al., 2024). To account for known sex differences in body composition and absolute energy turnover, whereby men typically exhibit higher body mass and lean body mass and consequently higher absolute energy expenditure during exercise, we additionally examined energy expenditure relative to body mass and lean body mass. This normalization approach may allow for a more physiologically meaningful comparison between sexes by reducing confounding effects of body mass and lean body mass, as differences in absolute energy expenditure have been shown to vanish when accounting for body composition (Archacki et al., 2024).

## 2 MATERIALS AND METHODS

### 2.1 Participants

Nineteen healthy endurance athletes (9 women and 10 men) were recruited by word of mouth and email. To meet the inclusion criteria, the participants were required to be between 18 and 40 years old, to have no chronic illness, and to be within the normal weight range of the BMI. They also had to be non‐smokers and not excessive alcohol drinkers. In addition, they had to be engaged in training for more than 7 h per week in endurance‐related sports, including triathlon, running, cycling, and swimming to ensure that they can sustain prolonged cycling up to 3 h. Male participants had to have a VO_2max_ ≥60 mL/min/kg, and female participants had to have a VO_2max_ ≥50 mL/min/kg. Participants were not included if they did not meet all the inclusion criteria or if they had any abnormalities of the cardiovascular system, as assessed by a resting electrocardiogram and examination by a physician. To avoid hormonal disturbances of the menstrual cycle, the test days for women were only conducted within the first 10 days of their menstrual cycle. This study adheres to the principles of the Declaration of Helsinki for use of human participants and tissues and was approved by the Medical Ethics Committee of the Technical University of Munich. Each participant gave written informed consent after being fully informed about the procedure and potential risks of the study.

### 2.2 Test protocol

Each included study participant had 3 days of visit. The timing of each test day was consistent, with blood sampling occurring at approximately the same time each day to minimize within‐day variation. The initial visit included the VO_2max_ test. Prior to a three‐minute warmup at 80 W, a resting phase of 3 min was obligatory to become familiar with the test setup. Following the warmup phase, the loading phase with an increase of 20 W/min started and ended solely at volitional exhaustion. Blood sampling was conducted prior to and directly after the VO_2max_ test. The individual ventilatory threshold 1 (VT1) was determined according to the data obtained via spiroergometry and validated via lactate measurements (Biosen S‐line Lab+, EKF Diagnostic, Cardiff, United Kingdom).

On the second visit, the participants were required to engage in 3 h of cycling in the moderate domain, previously determined by their individual threshold (VT1–5%). The participants arrived in a fasted state. One hour prior to the test, each subject was required to drink 500 mL of water with an individual amount of glucose powder, calculated as 1.5 g per kilogram body weight. We used this standardized glucose solution to harmonize energy intake, to reduce nutritional interference of different meals, and to prevent hypoglycaemia during prolonged exercise. Blood sampling was conducted at the following timepoints: 120 min prior to start (−2 h Pre), at start (Pre), and at 30‐min intervals throughout the test (30 min, 60 min, 90 min, 120 min, 150 min, and Post) as well as 60 and 120 min after cessation (1 h Post, 2 h Post). On the third visit, which was the day following the 3‐h cycling test, an additional blood sample was collected (24 h Post).

Cycling was performed on either a Lode Excalibur Cycling Ergometer (Lode B.V, Netherlands) or on a SRM cycling ergometer (SRM International, Germany). The oxygen uptake was measured with a Cortex Metalyzer® 3B and the corresponding software MetaSoft® Studio (CORTEX Biophysik GmbH, Germany). Carbohydrate and fat oxidation rates were calculated from gas exchange measurements using standard equations for substrate oxidation (Jeukendrup & Wallis, 2005). Energy expenditure was calculated using a 30‐s moving average. Lactate concentrations were measured with capillary blood using Biosen S‐Line Lab+ (EKF‐diagnostic GmbH, Barleben, Germany). The body composition was assessed using body impedance analysis with the InBody 270 device (InBody Europe B.V., Eschborn, Germany).

### 2.3 Blood sample collection

On the day of the performance analysis, venous blood samples were taken from the antecubital vein using standard needles (Safety‐Multifly® 21G tube 200mm, Sarstedt AG & CO. KG, Germany). This was done before and after the exercise test. During the 3‐h cycling test, multiple blood samples were obtained via a catheter (Venflon™ Pro Safety, G22, Becton Dickinson) in the forearm. Prior to the first blood sampling, the catheter was cleaned with 1 mL of sterile NaCl‐solution (0.9%). Moreover, the initial 2 mL of whole blood were discarded to remove residual NaCl‐solution from the catheter. At a minimum, the blood samples were collected using 2.7 mL monovettes (S‐Monovette® 2.7 mL K3EDTA, Sarstedt AG & Co. KG, Germany). After blood collection, the monovettes were inverted for 3 min and subsequently centrifuged for 10 min at 2460 × *g* and 20°C in a Hettich Mikro 22 centrifuge (Andreas Hettich GmbH & Co. KG, Germany). In a second step, the centrifuged plasma was pipetted into 250 μL aliquots (Eppendorf 1.5 mL DNALoBind Tubes, Hamburg) and centrifuged again for 10 min at 10,000 × *g* (Mini Spin Plus, Eppendorf, Germany) to remove residual solid blood components. The supernatant was transferred into a fresh tube, snap frozen on dry ice, and stored at −80°C until analysis.

### 2.4 Quantification of cfDNA

cfDNA was quantified using a direct quantification protocol that did not require DNA isolation, as described and validated by Neuberger, Brahmer, et al. (2021). This was conducted using an Assist Plus pipetting robot (Integra Biosciences GmbH, Biebertal, Germany). EDTA plasma samples were thawed in a water bath at 30°C for 10 min and diluted 1:15 using UltraPure DNase/RNase‐free H_2_O (Invitrogen, Waltham, MA, catalogue number 10977049). 2 μL of the dilution was mixed with 0.2 μL of primer mix (140 nM final concentration) and 7.8 μL of master mix to prepare technical duplicates with 5 μL final volume. The above‐mentioned primers were ordered from Eurofins Genomics (Ebersberg, Germany). The primer sequences were 5′‐TGCCGCAATAAACATACGTG‐3′ and 5′‐GACCCAGCCATCCCATTAC‐3′ priming a 90‐base pair fragment of DNA from the L1PA2 region. The final concentrations of the master mix components in the PCR reaction were as follows: 1.2 × Hifi Buffer (Bioline, catalogue number BIO‐21099‐BL), 0.3 mM dNTPs (Bioline, catalogue number BIO‐39043‐BL), 0.15 × SYBR Green nucleic acid gel stain 10,000 × (Sigma‐Aldrich, catalogue number C90M1158), and 0.04 u/μL Velocity Polymerase (Bioline, catalogue number BIO‐21099‐BL). The final dilution of the plasma in the PCR reaction was 1:75. Prior to initiating the qPCR with a Bio‐Rad CFX384 system (Bio‐Rad, Hercules, CA, USA), the plate was centrifuged at 1600 × *g* for 2 min. After 2 min at 98°C, 35 cycles were conducted. Each cycle consisted of 10 s at 95°C for denaturation and 10 s at 64°C for annealing. The melt curve analysis started at 70°C, with an incremental increase of 0.5°C every 10 s up to 95°C. The cfDNA concentrations (in ng/mL) were calculated according to Neuberger, Brahmer, et al. (2021).

### 2.5 Statistics

All statistical analyses were performed using R Statistical Software (v 4.2.2; R Core Team 2022). The anthropometric and cardiopulmonary data of the participants (Table 1) were compared using Welch's *t*‐tests, after validation of normal distribution using Shapiro–Wilk‐Test and variance homogeneity using Levene's test. The non‐normally distributed data of Watt at VT1–5% was compared using Wilcoxon Rank Sum Test. Within the text, all data are presented as mean ± standard deviation (SD). To identify differences in cfDNA kinetics at different time points in response to the VO_2max_ test or the 3‐h constant load test between male and female participants, linear‐mixed effects models were performed using lme4 (v 1.1–35.5) package, accounting for incomplete repeated measurements, with an overall data completeness of 94.6% of planned samples. The dependent variable was the log2‐transformed cfDNA. Time, sex, and their interactions were specified as fixed effects, while the subject was specified as random effect, as described by the following formula: log2(cfDNA)~time point * sex + (1 | subject). The significance of the model was determined with an *F*‐test implemented in the lmerTest (v 3.1–3) package. For post‐hoc comparisons Tukey's HSD tests with Bonferroni‐Holm correction were computed using the emmeans package (v 1.10.3). Correlations between cfDNA and other parameters were computed using Spearman rank correlations (ggpmisc v 0.6.0).

**TABLE 1:** Anthropometric and cardiopulmonary exercise testing related data of the participants. The differences between sex were computed using Welch's *t*‐tests. Differences for non‐normal distributed data (Power output at VT1–5%) were computed using Wilcoxon Rank Sum test.

Targets	Overall		Men		Women		Test results sex		
	*n*	Mean ± SD	*n*	Mean ± SD	*n*	Mean ± SD	Statistic	Df	*p*
Age [years]	18	31.0 ± 5.8	9	30.9 ± 6.1	9	31.1 ± 5.8	−0.08	15.94	0.9380
Height [cm]	18	173.0 ± 9.6	9	180.1 ± 6.1	9	165.9 ± 6.8	4.63	15.82	0.0003
Weight [kg]	18	67.7 ± 9.4	9	74.0 ± 6.8	9	61.4 ± 7.2	3.83	15.94	0.0015
Bodyfat [%]	18	17.3 ± 5.6	9	13.6 ± 3.4	9	20.9 ± 5	−3.60	14.25	0.0028
Resting heartrate [bpm]	17	52.7 ± 9.9	9	50.9 ± 12.0	8	54.8 ± 7.2	−0.82	13.29	0.4280
VO_2max_ [mL/min/kg]	18	58.4 ± 7.1	9	64.2 ± 4.2	9	52.7 ± 3.9	6.04	15.93	<0.0001
Peak power output [W]	18	366.1 ± 58.7	9	418.6 ± 22.6	9	313.6 ± 24.6	9.42	15.89	<0.0001
VO_2_ at VT1–5% [mL/min/kg]	18	35.1 ± 5.6	9	37.7 ± 4.4	9	32.5 ± 5.6	2.19	15.14	0.0448
Power output at VT1–5% [W]	18	184.2 ± 43.6	9	215.9 ± 33.8	9	152.5 ± 25.3	75		0.0027

To identify sex‐specific differences in cfDNA kinetics considering energy expenditure during the 3‐h constant load test, we computed linear mixed models between cfDNA differences from pre values, using the following formula: log2(cfDNA)~energy expenditure + sex + (1 | subject). Additionally, we computed non‐linear generalized additive mixed models (GAMMs) across all cfDNA values using the mgcv package (v 1.9–1): log2(cfDNA) ~ s(energy expenditure, by = sex) + sex + s(subject, bs = “re”). The energy expenditure was further adjusted for body mass or lean body mass. A *p* value <0.05 was considered statistically significant. All figures were illustrated using ggplot2 (v 3.5.1).

## 3 RESULTS

### 3.1 Study cohort

Of the 19 participants who were recruited for the study, one was excluded due to difficulties regarding blood sampling. Furthermore, the samples of the 3‐h cycling test of one subject were excluded due to flawed blood samples. In total, 16 participants (9 women and 7 men) were included in the analysis of the VO_2max_ test, while 17 participants were included in the statistical analysis of the 3‐h cycling test. Except for two women, who had a VO_2max_ of 48 mL/min/kg, each participant met all of the inclusion criteria. However, to ensure an equal number of female and male participants, the two women were included, despite their VO_2max_ being slightly below 50 mL/min/kg.

As shown in Table 1, the female and male participants were of similar age (women: 31.1 ± 5.8 years; men: 30.9 ± 6.1 years), but differed in respect to height, bodyweight, bodyfat (%), and relevant performance related parameters, including VO_2max_ and VO_2_ at VT1–5% (Table 1).

### 3.2 cfDNA in response to the VO_2max_ test

**FIGURE 1:**
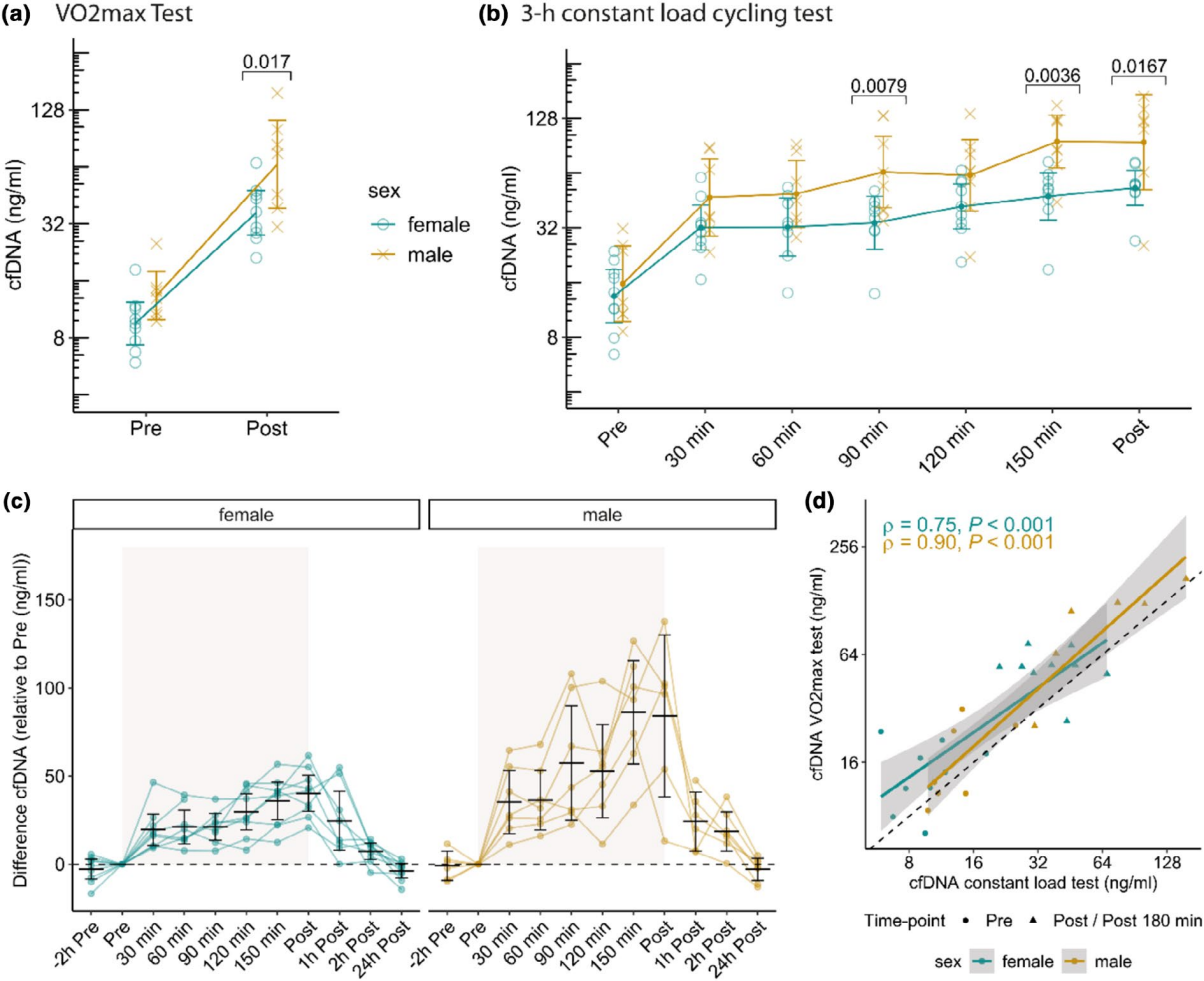
(a) cfDNA concentrations before and immediately after the VO_2max_ cycling test (*n* = 16). (b) cfDNA concentrations before, at the indicated timepoints and after the 3‐h constant load cycling test (*n* = 15–17 per timepoint). (c) differences in cfDNA concentration to the Pre‐measurements at the indicated timepoints during the 3‐h constant load cycling test (*n* = 15–17 per timepoint). The grey area indicates the period of constant load exercise. Mean and 95% CI are shown. (d) correlation of cfDNA concentrations between the constant load test and the VO_2max_ test (*n* = 32).

In response to the VO_2max_ test, the cfDNA concentrations increased 4.7‐fold (± 3.5) from 14 ± 5.2 at rest to 76 ± 43.9 ng/mL after exercise in men and from 10.1 ± 3.7 ng/mL to 38.7 ± 14 ng/mL in women, respectively. The cfDNA concentrations differed significantly between women and men (*F*_(1,14)_ = 6.628, *p* = 0.022). A Tukey HSD post‐hoc test revealed significantly higher cfDNA values in men than in women after exercise (*p* = 0.017). In contrast, resting cfDNA concentrations did not differ significantly (Figure 1a).

### 3.3 3‐h constant load cycling test

During the 3‐h cycling test, the overall cfDNA concentrations increased 4.9‐fold (± 3.5). In men, concentrations rose from 17.7 ± 9.2 at rest to 52.9 ± 27.4 ng/mL after 30 min, reaching 108.8 ± 48.7 ng/mL at the end of the exercise. In women, concentrations increased from 14.6 ± 5.9 ng/mL at rest to 34.2 ± 12.9 ng/mL after 30 min and 54.8 ± 13.5 ng/mL at the end of the exercise. A significant effect of timepoint (*F*(6, 84) = 75.49, *p* < 0.001), sex (*F*(1, 15) = 6.00, *p* = 0.027) and timepoint × sex interaction (*F*(6, 84) = 2.21, *p* = 0.050) was identified considering all values before (Pre), during (30 min, 60 min, 90 min, 120 min) and at termination (180 min) of the test with males exhibiting higher cfDNA levels overall. Tukey HSD post‐hoc tests revealed significant differences at 90 min, 150 min, and 180 min (Figure 1b). As shown in Figure 1c the differences of cfDNA in relation to Pre exercise indicate a rapid increase of cfDNA after 30 min and a second increase after 150 min.

Notably, we observed no change in blood lactate values. This provides evidence supporting that the participants were at a metabolic steady state and exercising within their moderate domain. Therefore, no significant changes over the course of the 3 h could be found. At onset of exercise, it was at 1.28 ± 0.27 mmol/L (men: 1.13 ± 0. 23 mmol/L; women: 1.17 ± 0.24 mmol/L) and changed only slightly to 1.25 ± 0.30 mmol/L (men: 1.34 ± 0.29 mmol/L; women: 1.16 ± 0.29 mmol/L) after 3 h of cycling.

#### 3.3.1 cfDNA concentration in relation to 3‐h cycling and VO_2max_ test

In comparison to the VO_2max_ test, the 3‐h constant load test resulted in higher cfDNA increases from pre to post (*p* = 0.0256), with mean differences of 43.2 (±32.4) ng/mL in response to the VO_2max_ test and 63.2 (±40.7) ng/mL in response to 3‐h constant load. After both tests, the cfDNA differences were higher in male compared to female participants (*p* = 0.003 and *p* = 0.002, respectively). The absolute concentrations of cfDNA correlated between the two tests for female and male participants with *R* = 0.75 and 0.90, *p* < 0.001, respectively.

Notably, in response to the VO_2max_ test the peak power output was significantly correlated with Post cfDNA concentrations (*r* = 0.63, *p* = 0.010), as well as with cfDNA differences (Post – Pre cfDNA) (*r* = 0.58, *p* = 0.020). After splitting the data into male and female participants, a significant correlation was detected for female participants (*r* = 0.73, *p* = 0.031), but not for male participants (*p* > 0.05), which might be related to the small sample size and a data outlier. Similarly, in response to the constant load, cfDNA values at Post and cfDNA differences correlated with the energy expenditure for the 3 h of cycling (*r* = 0.51, *p* = 0.040 and *r* = 0.57, *p* = 0.023, respectively), whereas significance disappeared after accounting for sex.

### 3.4 Clearance of cfDNA after the 3‐h constant load cycling test

In male and female participants, the concentration of cfDNA remained increased after 2 h of rest (*p* < 0.001, *p* = 0.002, respectively) compared to the Pre concentrations. Twenty‐four hours after finishing cycling, the values returned to baseline.

### 3.5 Sex differences of cfDNA in relation to energy expenditure

**FIGURE 2:**
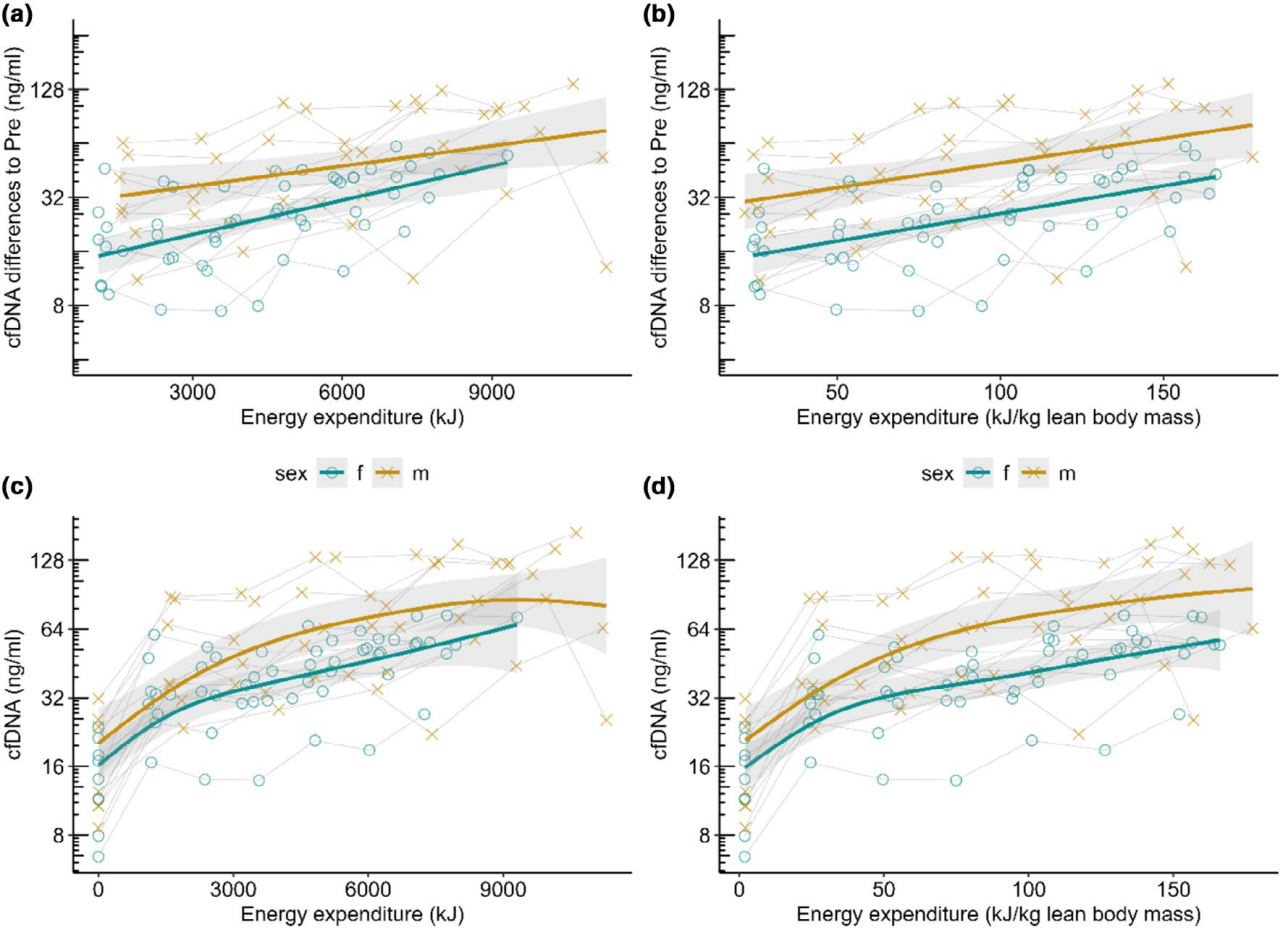
Linear relation of cfDNA differences to (a) energy expenditure (kJ) and (b) energy expenditure (kJ) per kg lean body mass (*n* = 90). Non‐linear relation of cfDNA concentration to (c) energy expenditure (kJ) and (d) energy expenditure (kJ) per kg lean body mass indicates sex differences in response to constant load cycling (*n* = 113). Solid lines represent fitted models, with gray areas indicating 95% confidence intervals.

To identify further sex differences in the cfDNA kinetics, we compared the differences in cfDNA concentration relative to energy expenditure (Figure 2a) and energy expenditure per kg body weight, or lean body mass (Figure 2b). Men showed consistently higher cfDNA responses than women, with effect estimates ranging from *β* = 0.69 to 0.94. The effect of sex difference was statistically significant when energy expenditure was normalized to body weight (*β* = 0.85 ± 0.35, *t* = 2.46, *p* = 0.028) or lean body mass (*β* = 0.94 ± 0.34, *t* = 2.78, *p* = 0.015). However, no significant effect was observed when absolute energy expenditure was considered (*β* = 0.69 ± 0.35, *t* = 1.94, *p* = 0.07). Normalizing for body composition slightly amplified the sex difference but did not improve overall model performance (ΔAIC <2; conditional *R* ^2^ = 0.77–0.79).

Similarly, computing non‐linear generalized additive mixed models across all cfDNA values, cfDNA concentrations increased non‐linearly with energy expenditure, as indicated by highly significant smooth terms for both sexes (*F* = 35–44, *p* < 0.001) and high overall model fits (adjusted *R* ^2^ = 0.87–0.88). When accounting for sex, the models revealed consistently higher cfDNA concentrations in men. When absolute energy expenditure was used as a predictor, the difference showed no significance (*β* = 0.47 ± 0.28, *t* = 1.65, *p* = 0.10). Normalizing energy expenditure to body mass (*β* = 0.61 ± 0.28, *t* = 2.22, *p* = 0.029) or lean body mass (*β* = 0.65 ± 0.27, *t* = 2.40, *p* = 0.019) strengthened and rendered the sex effect statistically significant (Figure 2d).

## 4 DISCUSSION

In the present study, we investigated the kinetics of cfDNA in response to a VO_2max_ test and in the moderate domain after 3‐h cycling at the individual ventilatory threshold one minus 5%. Our main findings are that (1) cfDNA increases are higher following 3‐h cycling than VO_2max_ test and (2) the cfDNA increases are significantly higher in endurance trained men compared to their trained female counterparts.

Exercise‐induced cfDNA releases depend on exercise modality, duration, and intensity (Breitbach et al., 2012; Haller et al., 2017; Nogiec et al., 2024). Like other studies, we observed ~4.7‐fold (men = 5.4‐fold, women = 3.8‐fold) increases in response to the VO_2max_ cycling test in endurance trained athletes. Previous studies on VO_2max_ cycling tests with healthy participants observed ~4.44‐fold (Tug, Mehdorn, et al., 2017), ~6.54‐fold (calculated from concentrations in Brahmer et al., 2019), and ~ 4.06‐fold (Neuberger, Hillen, et al., 2021) increases from pre‐ to immediately post‐exercise, respectively. Notably, Brahmer et al. and Tug et al. only included male athletes, and Neuberger et al. included just one female athlete. Furthermore, the VO_2max_ tests used in their studies followed a stepwise incremental protocol with three‐minute intervals, which results in longer cycling durations compared to the VO_2max_ test protocol used here.

In our study, the cfDNA levels increased ~4.9‐fold after the 3 h constant load cycling, with the highest increases occurring during the first 30 min of the exercise protocol, both in male and female participants. To our knowledge, only one study investigated the effect of constant load cycling. Sawai et al. (2024) studied the effect of 30 min cycling at 60% of VO_2max_ in female participants, three times at different menstrual cycle phases. Depending on menstrual phase, the authors measured cfDNA increases between 3.8‐ and 7.8‐fold (fold‐increase estimated based on figure 3 in Sawai et al., 2024). In our female group, we could show a 2.3‐fold increase after 30 min of cycling and 3.7‐fold increase after the whole exercise test, which is slightly lower. Differences could be caused by different intensities of the constant load because we used VT1–5%. Although the present load equals ~61.7% of VO_2max_, the differences could be a result of different exercise protocols in the VO_2max_ test. Furthermore, the mean aerobic capacity of our female group was higher (VO_2max_ = 52.7 mL/kg/min) than in the group of Sawai et al. (VO_2max_ = 45.3 mL/kg/min), and the phase of menstrual cycle might vary in the present study.

The circumstance that cfDNA increased independently of lactate during the 3‐h cycling test is in line with the literature. It has been speculated that lactate might have an impact on the accumulation of cfDNA (Beiter et al., 2011) but this was only shown in all‐out tests with several measurement points, where lactate and cfDNA rise in parallel at every stage. For exercises at low to moderate intensity (Breitbach et al., 2012; Haller et al., 2017) such as the conducted 3‐h cycling protocol in this study, rising levels of cfDNA due to exercise were independent of lactate.

During the 3‐h cycling bout, the highest increases in cfDNA occurred after the first 30 min. Importantly, cfDNA concentrations always reflect the interplay between cfDNA release and decay. The release of cfDNA during exercise is discussed to originate from neutrophils (Fridlich et al., 2023; Neuberger, Sontag, et al., 2021; Nogiec et al., 2024). The decay depends on circulating nucleases in the blood, including DNase I (Kustanovich et al., 2019). Velders et al. (2014) demonstrated enhanced DNase activity mediated by acute exercise. Enhanced DNase activity could be a reason for lower increases after 30 min as new release of cfDNA would then be counteracted by degradation. In addition, Ondracek et al. (2022) have shown that regular endurance exercise could increase DNase activity. In our study, we also recruited endurance trained athletes, indicating that high levels of physical performance could go along with higher DNase activity and so with maybe lower cfDNA concentrations.

Notably, the cfDNA concentrations returned slower to resting levels after the 3‐h cycling bout when compared to recovery after a VO_2max_ test. cfDNA typically shows a half‐life time of about 15 to 25 min, in all‐out exercise tests leading to a rapid return to resting levels after the end of an exercise protocol (Beiter et al., 2014; Haller et al., 2017; Neuberger, Sontag, et al., 2021; Tug, Mehdorn, et al., 2017; Yamamoto et al., 2025). In this study, the cfDNA levels were still higher 2 h after the exercise compared to the Pre values. Until now, this has only been shown in ultra‐marathon runners where the concentrations also stayed elevated 2 h after the race and only returned to baseline 24 h post (Atamaniuk et al., 2008). This might be related to a lower decay rate or a delayed or sustained effect of NETosis – the formation of neutrophil extracellular traps during which cfDNA is released (Thiam et al., 2020) – which might only become apparent following prolonged exercise.

Overall, male athletes had higher cfDNA concentrations than female athletes after the VO_2max_ test and throughout the 3‐h cycling test with significantly higher levels at several measurement timepoints. Importantly, this difference cannot be explained by greater load, as the exercise protocol was standardized relative to individual thresholds. To further examine whether differences in metabolic cost might account for the observed sex effect, we additionally performed an adjustment for energy expenditure. Although this analysis was not powered as a confirmatory test, the difference persisted, indicating that the higher cfDNA concentrations in males cannot be solely attributed to increased energy expenditure. This expands on the findings by Blumkaitis et al. (2024) who also observed greater cfDNA increases in men compared to women. In their study, however, the differences emerged in all‐out running tests where male participants had a longer total exercise duration, higher lactate concentration, and higher relative peak power output. Nogiec et al. (2024) likewise detected significantly higher cfDNA concentrations in their male participants in endurance testing, but when normalizing for expended calories, there was only a trend towards a significant difference. In our cohort, normalization of cfDNA to energy expenditure relative to body mass and lean body mass similarly suggested that the sex difference persists. Notably, Nogiec et al. studied a smaller cohort (6 men, 6 women) with lower VO_2max_ (~45 mL/min/kg) compared to our study.

There are several factors which could potentially contribute to the more pronounced cfDNA increases observed in men. As neutrophils are considered the main source of cfDNA in exercise, sex‐related differences in neutrophil characteristics warrant closer examination. Neutrophils in men and women seem to differ in counts, activation patterns, propensity of NETosis, and overall reactivity (Bain, 1996; Gupta et al., 2020; Lu et al., 2021). In women, neutrophil activity may also vary across different phases of the menstrual cycle, likely due to fluctuating hormone levels (Smirnova et al., 2018). To minimize the impact of hormonal variation in our study, all exercise tests involving female athletes were conducted within the first 10 days of their menstrual cycle when hormonal fluctuations are relatively low. While this approach aimed to control for cycle‐related variability, previous studies also suggest that cfDNA concentration in women may not be significantly influenced by menstrual cycle phase (Pölcher et al., 2010; Sawai et al., 2024).

Although some studies suggest that women generally exhibit higher neutrophil activity and prolonged neutrophil survival under the influence of estradiol and progesterone (Molloy et al., 2003; Richter et al., 2025), cfDNA increases appeared more pronounced in men. This discrepancy is supported by evidence that males show higher baseline NET markers and circulating NETs, possibly due to increased ROS production and impaired clearance mechanisms, whereas female neutrophils demonstrate an enhanced but more controlled NET release upon stimulation (Richter et al., 2025). This highlights that sex‐specific modulation of neutrophils and NETosis is not yet fully understood and differences in cfDNA responses to exercise cannot be fully explained by neutrophil activity alone. Moreover, absolute cell counts and blood volume may also influence cfDNA concentrations to an unknown extent, but these factors were not assessed in our study and, to our knowledge, have not been investigated in previous exercise studies on cfDNA. Precise estimation would require time‐point–specific measurements for each participant, which is methodologically challenging, but could provide valuable insights into sex differences in cfDNA. Future studies should therefore aim to investigate the tissue of origin, neutrophil function including elastase activity, and hormonal influences in more detail, and consider including absolute cell counts and blood volume estimation.

In addition, differences in substrate oxidation could represent another potential factor contributing to sex‐specific responses. Aligning with previous literature (Cano et al., 2022), male participants exhibited higher RER values during the prolonged cycling protocol despite matched relative exercise intensity, reflecting higher carbohydrate oxidation compared to the female participants. Increased carbohydrate oxidation is associated with enhanced glycolytic flux, which has been shown to modulate immune activation and inflammatory responses (Llibre et al., 2025). Therefore, these differences may reflect subtle variations in metabolic stress between sexes, although the present study was not designed to directly assess their relationship with cfDNA kinetics. Thus, the RER possibly could serve as a surrogate marker of greater metabolic stress in the male participants, but we did not find any established evidence that RER itself independently predicts cfDNA release.

A limitation of this study is that it does not distinguish between the cfDNA release and decay. Since we did not measure the activity of different DNases at the different timepoints, it is only possible to hypothesize how the interplay of cfDNA release and degradation results in the measured concentrations. Future studies should therefore additionally measure the concentrations of circulating enzymes which are involved in the degradation of cfDNA such as DNase I, plasma factor VII‐activating protease and factor H (Kustanovich et al., 2019). This will provide a further understanding of the kinetics of cfDNA during long‐term exercise but also the delayed return to baseline after exercise which is observed in this study. In addition, prolonged or delayed occurrence of NETosis should be analyzed to support time‐delayed effects of exercise.

Another limitation concerns the small sample size. No a priori power analysis was conducted; significant effects were observed for the main endpoints, suggesting that the study was sufficiently sensitive to detect the expected primary effects. In contrast, several additional analyses were conducted in an exploratory manner, particularly when stratifying by sex. The reduced sample size in these subgroup analyses likely limited statistical power, which may explain why some associations did not reach statistical significance despite showing similar trends. For example, we observed significant correlations between changes in cfDNA and energy expenditure in the full cohort, consistent with findings by Nogiec et al. (2024). However, these associations did not remain statistically significant in the male subgroup once the sample was split by sex. This likely reflects reduced statistical power rather than the absence of a true effect. Moreover, patterns indicative of sex‐specific associations with energy expenditure were apparent, but did not consistently reach statistical significance, suggesting that small‐to‐moderate differences may not have been detectable with the available sample size. Therefore, these findings should be regarded as hypothesis‐generating. Future studies with larger cohorts will be necessary to confirm potential sex‐specific relationships between energy expenditure and cfDNA release and to improve generalisability.

Another aspect is that, although all participants were endurance trained, their familiarity with cycling could have varied within the cohort. It remains unclear whether this could have influenced cfDNA responses, as evidence on the effect of training status is inconsistent, with studies reporting both differences and no differences between trained and untrained individuals (Beiter et al., 2014; Huminska‐Lisowska et al., 2021). Therefore, potential influences within our sample cannot be excluded, and the generalisability of the findings to untrained populations may be limited.

While we generally tried to ensure that all female test participants were in the same phase of the menstrual cycle – namely in the initial phase to ensure the least hormonal fluctuations – we did not carry out ovulation tests. In future studies, ovulation and hormonal status should be tracked precisely to prevent any potential influences on cfDNA release. While Sawai et al. (2024) did not identify significant effects of the menstrual cycle on the release of cfDNA, the observed trend requires further consideration.

## 5 CONCLUSION

This is the first study to investigate the kinetics of cfDNA during and after a long‐term aerobic load, showing that the highest increases in cfDNA concentration occur within the first 30 min. This study demonstrates that cfDNA concentrations in physical exercise show sex‐specific differences in VO_2max_ and 3‐h cycling protocols in endurance trained athletes. In addition to previous study results, secondary outcome analyses suggested that these differences may persist even when accounting for energy expenditure relative to body mass and lean body mass. The participants showed a delayed return to baseline cfDNA concentrations. Further investigations of enzymatic activity such as DNases, NETosis, and hormonal status should be conducted to gain a deeper understanding of the underlying mechanisms leading to sex‐specific differences and delayed degradation.

## AUTHOR CONTRIBUTIONS

**Kira Enders:** Data curation; formal analysis; investigation; visualization. **Moritz Dollsack:** Data curation; formal analysis; investigation. **Daniela Schranner:** Conceptualization; data curation; formal analysis; investigation; methodology; project administration. **Stephanie Bremer:** Formal analysis; investigation. **Perikles Simon:** Conceptualization; supervision. **Henning Wackerhage:** Conceptualization; funding acquisition; supervision. **Elmo W. I. Neuberger:** Conceptualization; formal analysis; methodology; visualization. **Martin Schönfelder:** Conceptualization; investigation; methodology; project administration; supervision.

## FUNDING INFORMATION

Research was funded by the German Federal Institute of Sport Science (ZMVI4‐070104/20‐21). Daniela Schranner was supported by a doctoral scholarship from the German National Scholarship Foundation (Studienstiftung des deutschen Volkes).

## CONFLICT OF INTEREST STATEMENT

The authors declare that they have no competing interests.

## ETHICS STATEMENT

This study adheres to the principles of the Declaration of Helsinki for use of human participants and tissues and was approved by the Medical Ethics Committee of the Technical University of Munich.

## CONSENT

Each participant gave written informed consent after being fully informed about the procedure and potential risks of the study.

## Data Availability

The data that support the findings of the current study are available from the corresponding author on request.
